# Early remission with rituximab in pediatric pemphigus foliaceous

**DOI:** 10.1016/j.jdcr.2025.06.067

**Published:** 2025-10-08

**Authors:** Reshma Gupte, Asharbh Raman

**Affiliations:** Department of Dermatology, Venereology and Leprosy, Dr. D. Y. Patil Medical College, Hospital and Research Centre, Dr. D. Y. Patil Vidyapeeth, Pune, Maharashtra, India

*To the Editor:* We read “Pediatric pemphigus herpetiformis treated with rituximab” by Hamed et al with great interest.^1^ The case reports a rare occurrence successfully controlled by rituximab, after attempts to taper corticosteroids caused disease relapse. While rituximab is Food and Drug Administration approved for pemphigus vulgaris in adults, studies have shown its safety and effectiveness in pediatric patients as well.^2^, ^3^, ^4^, ^5^ Desmoglein 1 (Dsg 1) levels were not measured by the author, which could have predicted disease severity and guided treatment with early rituximab intervention.

We recently came across a case of pediatric pemphigus foliaceous in a 12-year-old boy with extensive erosions and crusted adherent scaly plaques, concentrated over the face, ears, scalp, neck, and trunk (Fig 1, Fig 2, Fig 3). Mucosae were not involved. Histopathology revealed intraepidermal acantholytic bullae with lymphocytic dermal infiltrate (Fig 4). Dsg 1 levels were significantly raised (>200 RU/mL), confirming the diagnosis. Prednisolone (1 mg/kg/day) and azathioprine (50 mg/day) prescribed over a month showed minimal improvement. Similar to the reported case, remission was eventually achieved with 2 sequential doses of rituximab 375 mg/m^2^, administered 2 weeks apart.

**Fig 1:**
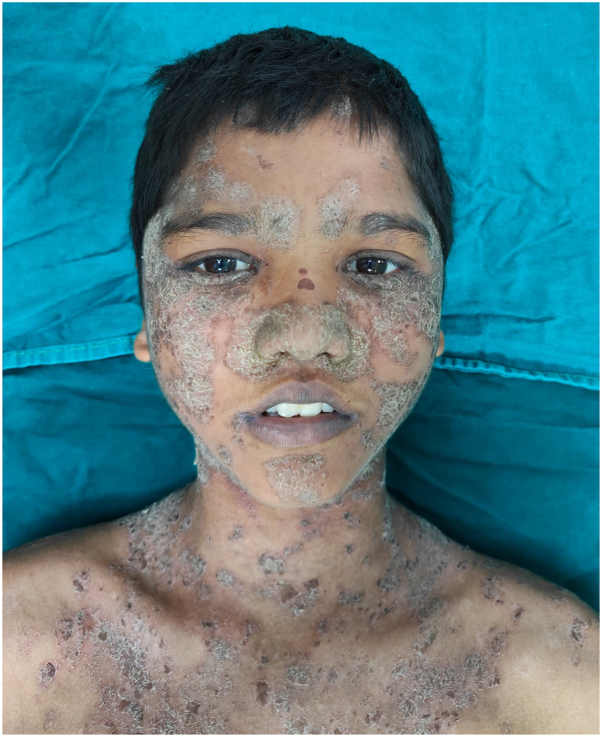
Multiple erosions and crusted scaly plaques over face, scalp, neck, and upper chest.

**Fig 2:**
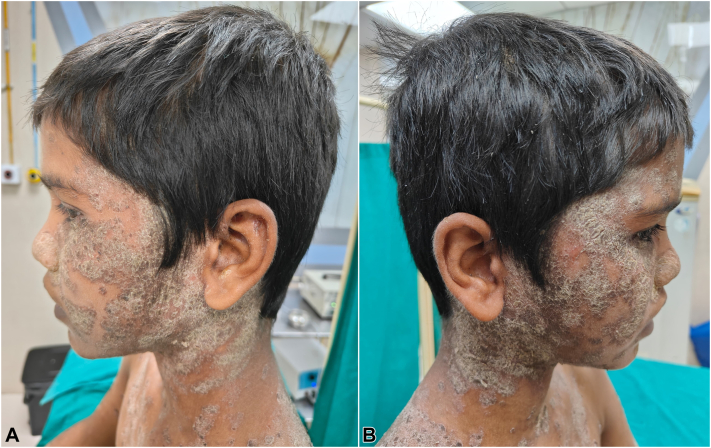
Multiple crusted scaly plaques over: **(A)** left half of the face, ear, and neck; **(B)** right half of the face, ear, and neck.

**Fig 3:**
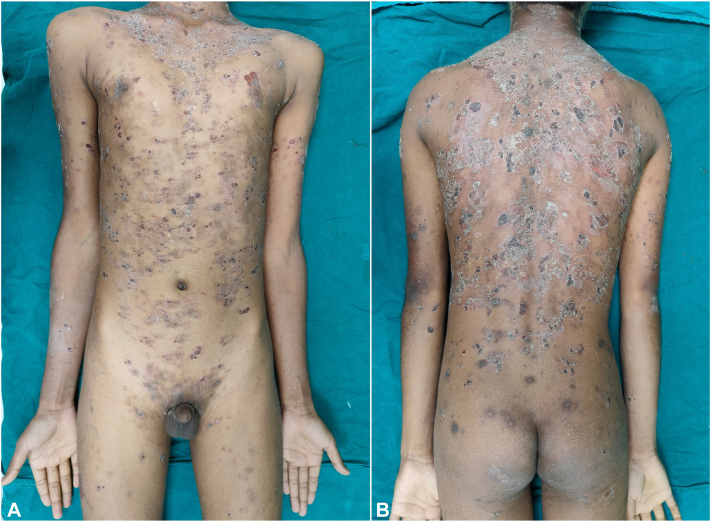
Multiple erosions and crusted scaly plaques over: **(A)** chest, abdomen, and anterior aspect of arms and thighs; **(B)** back, buttocks, and posterior aspect of arms.

**Fig 4:**
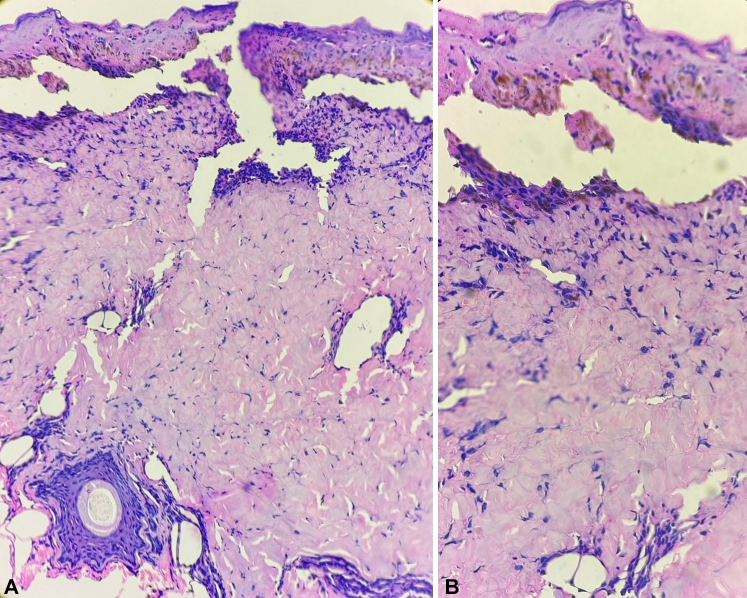
Histopathological examination showing intraepidermal acantholytic bulla with sparse dermal lymphocytic infiltrate [hematoxylin and eosin, **(A)** 40×; **(B)** 100×].

Early administration of rituximab is associated with a better long-term outcome and prolonged remission.^6^, ^7^, ^8^, ^9^ Yuval et al recommend it as a first-line treatment, owing to a better side effect profile compared to long-term corticosteroid usage, especially in the pediatric population.^5^ High Dsg 1 levels should prompt clinicians to intervene early with rituximab rather than waiting for the disease to become recalcitrant. Dsg 1 levels on follow-up can additionally guide maintenance therapy if required.

## Conflicts of interest

None disclosed.
